# Preparing for Longevity: Consumer Perspectives on Financial Preparedness, Risks, and Decision-Making

**DOI:** 10.1093/geroni/igaf122.1986

**Published:** 2025-12-31

**Authors:** Lauren Cerino, Lisa D’Ambrosio

## Abstract

As Americans live longer, financial preparedness is increasingly critical for ensuring stability and independence in later life. However, individuals’ perceptions of financial planning timelines, risk management products and strategies, and overall preparedness vary widely based on factors such as age, life stage, and income. Understanding these differences is essential for designing effective tools, policies, and interventions that support long-term financial security. This symposium presents findings from three MIT AgeLab studies exploring key aspects of financial decision-making across the lifespan. The first presentation explores how individuals at different life stages perceive the ideal timeframe for retirement preparation, comparing prospective (i.e., pre-retirement) and retrospective (i.e., post-retirement) insights on planning windows. The second presentation examines consumer attitudes, knowledge, and usage of financial products such as annuities and life and disability insurance designed to hedge against different financial risks. The third presentation explores how financially prepared middle-income Americans feel for later life, including the planning they engage in, and what kinds of opportunities or supports would support their future security. Together, these studies offer a comprehensive view of how people envision financial security, navigate risks, and engage with financial tools and products to support their later-life wellbeing. The session will provide valuable insights into the evolving landscape of longevity planning, followed by a discussion addressing implications for financial professionals, policymakers, and researchers in gerontology.

